# A zebrafish model of Poikiloderma with Neutropenia recapitulates the human syndrome hallmarks and traces back neutropenia to the myeloid progenitor

**DOI:** 10.1038/srep15814

**Published:** 2015-11-02

**Authors:** Elisa A. Colombo, Silvia Carra, Laura Fontana, Erica Bresciani, Franco Cotelli, Lidia Larizza

**Affiliations:** 1 Dipartimento di Scienze della Salute, Università degli Studi di Milano, Milan, Italy; 2 Dipartimento di Bioscienze, Università degli Studi di Milano, Milan, Italy; 3 Oncogenesis and Development Section, National Human Genome Research Institute, National Institutes of Health, Bethesda, MD, USA; 4 Laboratorio di Citogenetica Medica e Genetica Molecolare, IRCCS Istituto Auxologico Italiano, Milan, Italy

## Abstract

Poikiloderma with Neutropenia (PN) is an autosomal recessive genodermatosis characterized by early-onset poikiloderma, pachyonychia, hyperkeratosis, bone anomalies and neutropenia, predisposing to myelodysplasia. The causative *C16orf57/USB1* gene encodes a conserved phosphodiesterase that regulates the stability of spliceosomal U6-RNA. The involvement of *USB1* in splicing has not yet allowed to unveil the pathogenesis of PN and how the gene defects impact on skin and bone tissues besides than on the haematological compartment. We established a zebrafish model of PN using a morpholino-knockdown approach with two different splicing morpholinos. Both *usb1*-depleted embryos displayed developmental abnormalities recapitulating the signs of the human syndrome. Besides the pigmentation and osteochondral defects, *usb1*-knockdown caused defects in circulation, manifested by a reduced number of circulating cells. The overall morphant phenotype was also obtained by co-injecting sub-phenotypic dosages of the two morpholinos and could be rescued by human *USB1* RNA. Integrated *in situ* and real-time expression analyses of stage-specific markers highlighted defects of primitive haematopoiesis and traced back the dramatic reduction in neutrophil myeloperoxidase to the myeloid progenitors showing down-regulated *pu.1* expression. Our vertebrate model of PN demonstrates the intrinsic requirement of *usb1* in haematopoiesis and highlights PN as a disorder of myeloid progenitors associated with bone marrow dysfunction.

Poikiloderma with Neutropenia, Clericuzio-type (PN; OMIM#604173) is a rare autosomal recessive genodermatosis affecting the skin and the haematopoietic system, in particular the myeloid lineage. It is characterized by early onset poikiloderma, a cutaneous lesion with a pattern of reticulated hypo-hyper-pigmentation, mild atrophy and telangiectasias, pachyonychia, palmo-plantar hyperkeratosis, bone alterations and non-cyclic neutropenia, usually revealed by recurrent infections during infancy and childhood^1^,^2^,^3^,^4^,^5^. Discovery of the causative *C16orf57* gene by autozygosity mapping and next-generation sequencing of the linkage region on chromosome 16^6^, confirmed that PN is genetically distinct from clinically overlapping entities, in particular Rothmund-Thomson syndrome (OMIM#268400), and allowed the introduction of the genetic test to validate diagnosis and provide patients with the appropriate oncological surveillance^7^,^8^,^9^,^10^,^11^. Neutropenia, the distinctive PN hallmark, confers on patients a high risk to develop myelodysplasia, often evolving into acute myeloid leukaemia in the second decade of life^12^,^13^.

The *C16orf57* gene, renamed *USB1* (U six biogenesis 1), is phylogenetically conserved and ubiquitously expressed, suggesting a housekeeping function^6^,^12^.

Computational analysis of the encoded protein shows two conserved H-X-S/T-X tetra-peptide motifs (2H), which mark the active site of a 2-fold pseudosymmetric structure^5^. The signature of the 2H motifs, characteristic of the 2H phosphoesterase family^14^,^15^, suggested that the USB1 protein was likely to be involved in RNA processing, leading to the prediction that all the reported *USB1* loss-of-function mutations cause disruption of both protein folding and catalytic site^5^.

Recently, it has been demonstrated that in *Saccharomyces cerevisiae* and human cells the *USB1* gene and its yeast ortholog encode a phosphodiesterase that is essential for the biogenesis of the RNA splicing apparatus as it regulates the stability of U6 small nuclear RNA (snRNA)^16^,^17^. X-ray crystallography of the human USB1 protein has definitely confirmed that USB1 is an RNase that trims the 3′ end of the U6 transcript implicating aberrant oligoadenylation of U6 snRNA in the pathogenesis of PN^18^. While these insights advance our understanding of the PN pathomechanism, how *USB1* mutations impact on the morphogenesis and differentiation of tissues affected in PN remains unknown.

To address this issue, we have exploited the zebrafish as a model organism. Zebrafish has a single *USB1* ortholog, which is ubiquitously expressed during early development. Using a morpholino (MO) knockdown approach, we show that the morphants display developmental abnormalities that reproduce the signs of the human syndrome, namely defective pigmentation, osteochondral alterations and severe haematopoietic defects, mainly affecting the myeloid lineage.

## Results

### Identification and characterization of the zebrafish *usb1* gene

We performed BLAST searches with the human *USB1* gene and protein sequences to identify the zebrafish ortholog in the database (*usb1*, NM_001003460.1), which is present in only one copy in the zebrafish genome.

**Figure 1:**
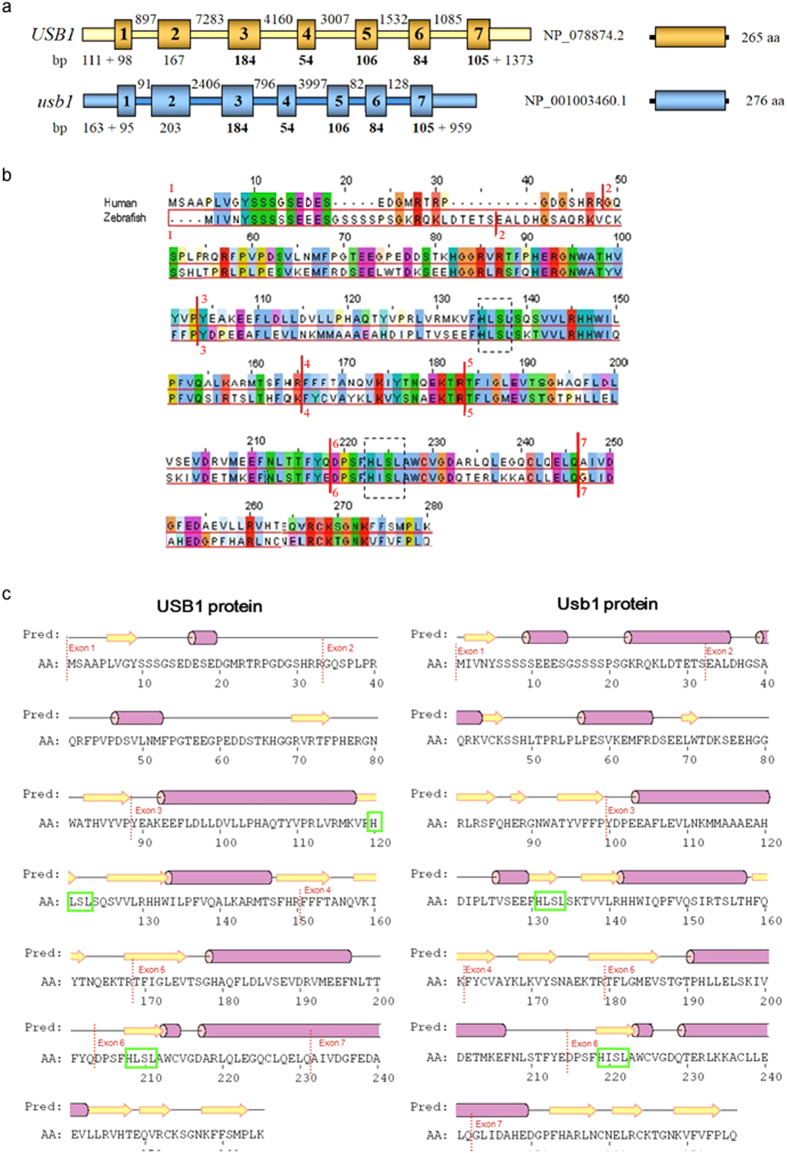
The human *USB1* gene, the zebrafish *usb1* ortholog and the encoded proteins. (**a**) Schematic genomic structure of *USB1* and *usb1*. The lengths, not to scale, of exons (boxes), UTRs and introns (interconnecting bars) are indicated below and over the scheme, respectively. The encoded proteins share 73.4% and 46% sequence similarity and identity, respectively. (**b**) BLAST alignment of the human (NP_078874.2) and zebrafish (NP_001003460.1) usb1 proteins. Identical amino-acid residues and residues with the same polarity can be noted in several positions. Vertical dotted lines highlight the conserved intron–exon junctions with flanking numbers denoting exon number. The two histidine-serine residues of the HLSL motifs that are a main part of the catalytic site of the protein^5^,^16^ are boxed. (**c**) Prediction of the secondary usb1 protein structure using the PSIPRED server (http://bioinf.cs.ucl.ac.uk/psipred/). Barrels represent the α-helices and arrows the β-strands. The two histidine-serine residues of the HLSL motifs are boxed.

The genomic structure of *USB1* and *usb1* orthologs is similar, with 7 exons in both cases (Fig. 1a) and the *USB1*- and *usb1*-containing regions on human chromosome 16q21 and zebrafish chromosome 25 are fairly syntenic.

The encoded human and zebrafish proteins are 265 and 276 amino acids (aa) in length, respectively, with a sequence similarity and identity of 73.4% and 46%, respectively. The difference in length between the genomic *usb1* sequence (9452 bp) and that of *USB1* (22072 bp) is accounted for by shorter *usb1* introns (Fig. 1a).

Alignment of the entire human and zebrafish protein sequences shows a highly evolutionarily conserved amino acids sequence from the 51st to the last aa residue (Fig. 1b).

The zebrafish protein shares with the human protein the distinctive series of secondary structure elements (α-helices and β-strands) and has the two HLSL motifs (boxed in Fig. 1b,c), which are the main part of the catalytic site in the human protein^5^,^16^,^17^,^18^.

### Expression profile of *usb1* during zebrafish development

**Figure 2:**
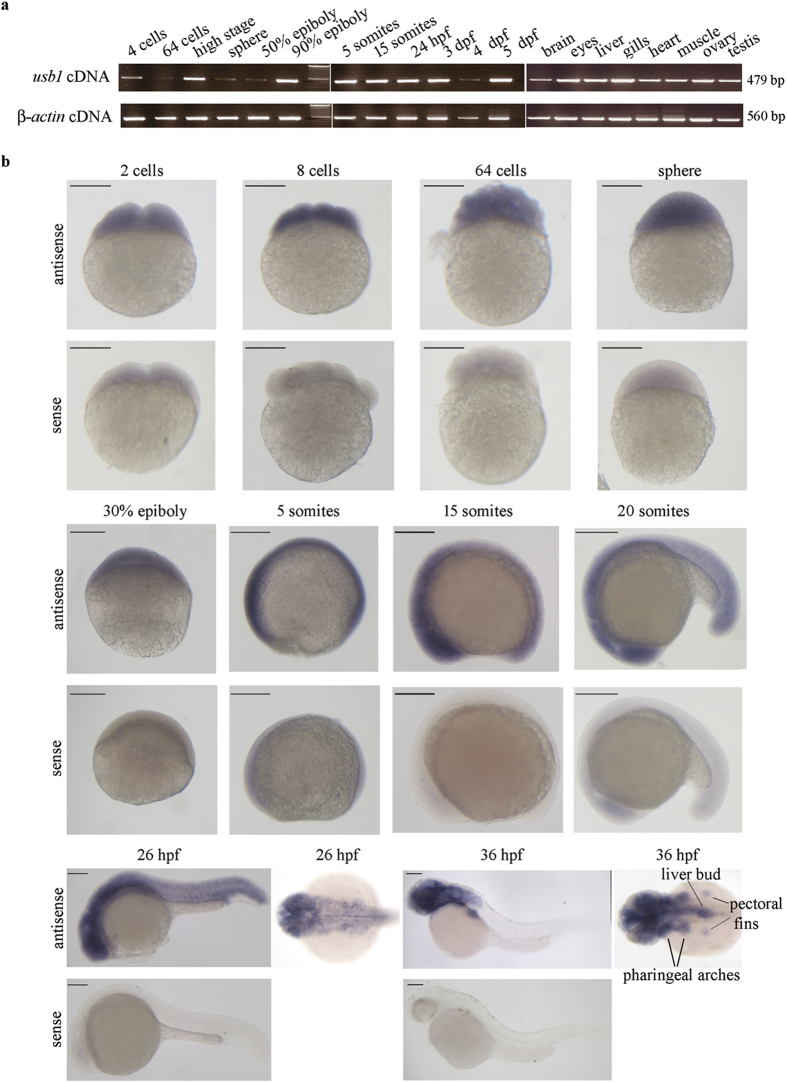
Expression profiling of *usb1* in zebrafish. (**a**) Temporal and spatial *usb1* expression during embryogenesis and among adult tissues. Total RNA was extracted from embryos at the indicated time-points and from the specified adult tissues, and subjected to RT-PCR with primers specific for *usb1* (upper panel) or *β-actin* (lower panel). The sizes of the obtained PCR fragments are indicated. (**b**) WISH analysis of *usb1*. The signal obtained with *usb1* antisense probe was ubiquitous until the 5-somite stage. It showed a higher intensity in the cephalic region at the 15 somites stage and displayed decreasing expression from the head to the caudal region (20 somites and 26 hpf). At 36 hpf, the signal only marked the head and the pharyngeal arches-derived structures. hpf: hour post fertilization; dpf: day post fertilization. Images showing the results obtained with sense probe are provided at each developmental stage. Scale bars: 200 μm.

Analysis of *usb1* expression during zebrafish embryonic development by reverse transcription-PCR (RT-PCR) showed that *usb1* transcript was both maternally and zygotically expressed (Fig. 2a). In adult animals, *usb1* expression was recorded in all analysed tissues (brain, eyes, liver, gills, heart, muscle, ovary and testis) (Fig. 2a).

Whole-mount *in situ* hybridization analysis (WISH) with an antisense probe targeting the full-length transcript (1,126 bp) was also used to characterize *usb1* expression patterns during embryo development (Fig. 2b). At early developmental stages, from two cells up to the 5 somites stage, *usb1* expression was ubiquitous and consistent with the expression found by RT-PCR (Fig. 2a). By the 15 somites stage until 26 hours post fertilization (hpf), expression of *usb1* was higher in the anterior head region than in the posterior tail (Fig. 2b). From 26 hpf, *usb1* transcripts were detected in the cephalic region and in specific trunk regions that at 36 hpf were recognized as the pharyngeal arches, the budding liver and the pectoral fins, while no expression was observed in the caudal region (Fig. 2b). At 36 hpf the expression persisted only in the anterior head and trunk regions (Fig. 2b). No signal was observed in embryos at the same developmental stage when stained with *usb1* sense probe (Fig. 2b).

### Design and validation of splice-blocking morpholinos

To investigate the role of *usb1* during zebrafish embryogenesis, we performed loss-of-function experiments using MO-mediated gene knockdown.

**Figure 3:**
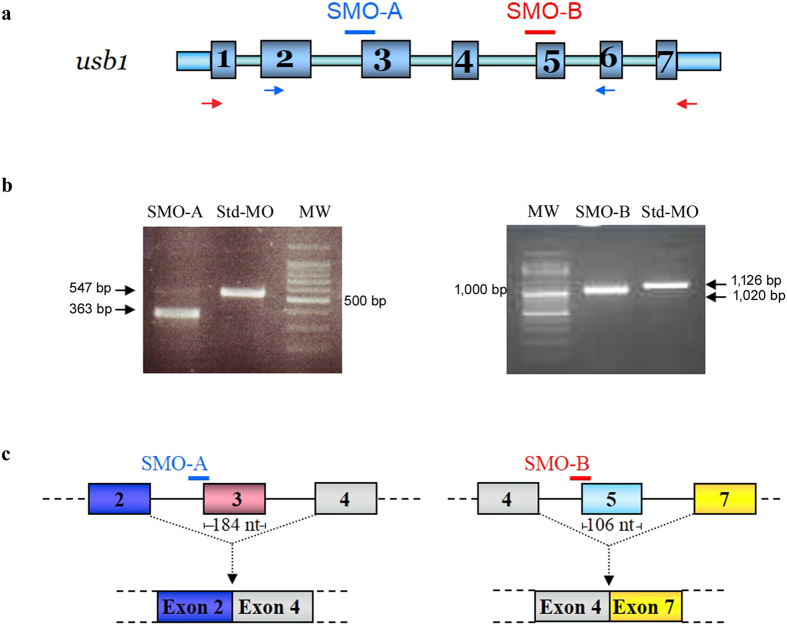
Efficiency of *usb1* knockdown by splice-blocking morpholino SMO-A, targeting IVS2 acceptor splice site, and SMO-B, blocking IVS4 acceptor splice site. (**a**) Schematic diagram of zebrafish *usb1* gene organization and position of SMO-A (blue) and SMO-B (red). Blue and red arrows indicate the positions of primer pairs used to detect the presence of aberrantly spliced *usb1* transcripts obtained with SMO-A (primers F2-R3, Supplementary Table S1) and SMO-B (primers F-R2, Supplementary Table S1), respectively. (**b**) RT-PCR analysis of *usb1* transcripts from embryos injected with SMO-A, SMO-B or Std-MO (each at 0.6 pmol/embryo). SMO-A morphants exhibited aberrantly spliced products (363 bp) as compared to Std-MO-injected embryos (547 bp) due to skipping of exon 3 (184 bp), frameshift, and exposure of a stop codon 24 nucleotides downstream. SMO-B morphants exhibited aberrantly spliced products (1020 bp) as compared to Std-MO-injected embryos (1126 bp) due to skipping of exon 5 (106 bp) following the block of the IVS4 acceptor splice site. MW, molecular weight markers. (**c**) Schematic representation of SMO-A- and SMO-B-mediated misspliced transcripts inferred by sequencing. Aberrant proteins are predicted in both cases as the 107 aa SMO-A-translated protein should lack both HLSL motifs, while the 184 aa SMO-B-translated protein should lack the second HLSL motif.

We injected two different splice-blocking MOs, SMO-A and SMO-B, targeting the *usb1* sequence between the IVS2 splice acceptor site and exon 3, and between the IVS4 splice acceptor site and exon 5, respectively (Fig. 3a).

Specific effects of SMO-A and SMO-B on *usb1* mRNA splicing were confirmed by RT-PCR, which showed a severe reduction in the amount of normal splice products and the formation of aberrant transcripts (Fig. 3b).

Sequence analysis revealed that the SMO-A misspliced amplicon of 363 bp was characterized by the out-of-frame skipping of exon 3. The corresponding aberrant transcript should result in a truncated protein (107 aa instead of 276 aa) lacking both HLSL motifs and therefore likely to be non-functional as the HLSL motifs are essential for the enzymatic function of the protein (Fig. 3c). The SMO-B aberrant transcript skipped exon 5, resulting in a protein that was missing the second of the two functionally relevant HLSL motifs (Fig. 3c).

### Overall phenotype of *usb1*-morphants

**Figure 4:**
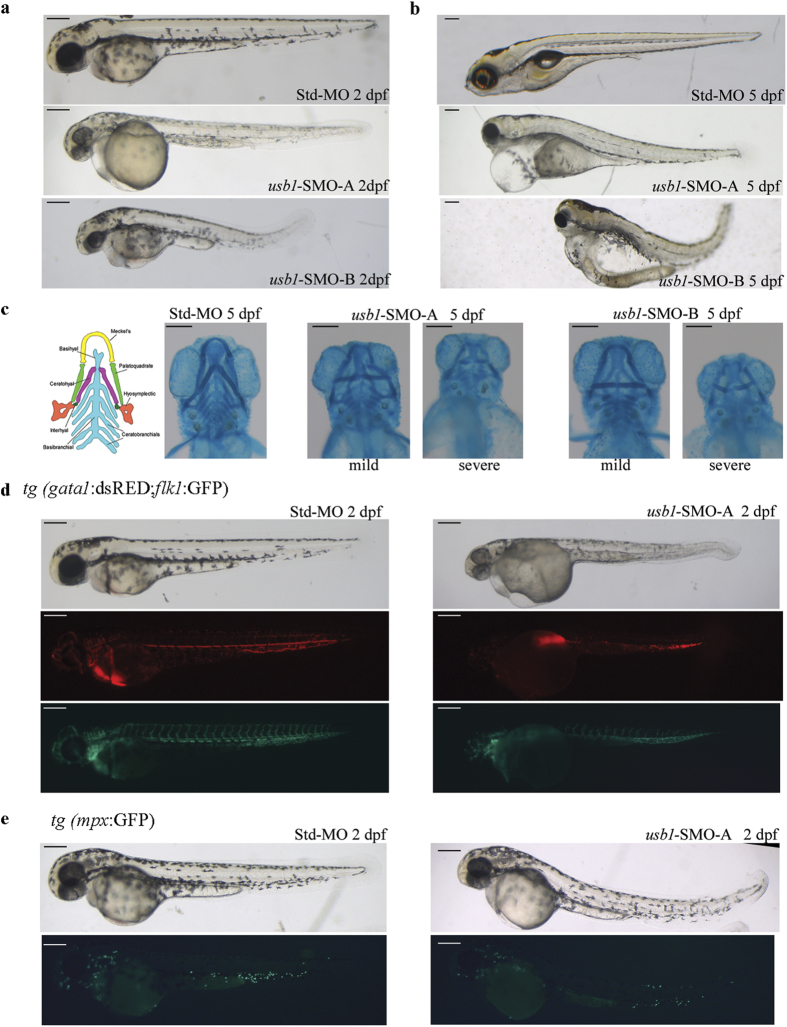
Overall phenotype of *usb1* morphants. Representative live-microscopy-4X magnification-pictures of 2 dpf (**a**) and 5 dpf (**b**) embryos injected with the same dosage (0.6 pmol/embryo) of Std (top), SMO-A (central) or SMO-B (bottom) morpholinos. The morphants display a smaller size, with defective and irregular pigmentation of the skin, as compared to the continuous stripes of the control embryos, and pericardial oedema. (**c**) Massive skeletal defects revealed by Alcian blue staining in *usb1* morphants at 5 dpf. From left to right: diagram illustrating the normal pharyngeal arches-derived bone architecture^19^; ventral view of a control embryo (left panel) and representative pictures of the mild (left) and severe (right) phenotypes of SMO-A and SMO-B morphants. The Meckel’s (yellow in the colour-coded diagram), the palatoquadrate (green) and ceratohyal (purple) structures appear misshaped in both mildly- and severely-affected embryos; moreover, the ceratobranchial structures (light blue) appear disorganized or missing in half of SMO-A and in a third of SMO-B severely-affected embryos. (**d**) Reduction of *gata1*-positive cells (red fluorescence) (middle panels) and a regular vasculature pattern (green fluorescence) (lower panels) in 2 dpf *tg(gata1:dsRED;flk1:GFP)* embryos injected with SMO-A (0.7 pmol/embryo) (right) as compared to control (left). (**e**) Myeloid lineage defects evinced by a reduction in *mpx*-positive cells (green fluorescence) (lower panels) in 2 dpf *tg(mpx:GFP;lyzC:dsRED)* embryos injected with SMO-A (0.7 pmol/embryo) (right) as compared to control (left). Scale bars: (**a–e**) 200 μm.

Following injection of SMO-A or SMO-B (each at a dosage of 0.6 pmol/embryo) into 1–2-cell embryos, specific defects could be observed in a consistent percentage of both morphant embryos at two (Fig. 4a) and five days post-fertilization (dpf; Fig. 4b) as compared to control embryos injected with the control MO (Std-MO) (Table 1).

At 2 dpf, most of SMO-A morphants appeared smaller than the controls, with smaller head, and 80% had pericardial oedema. Interestingly, 58% of the SMO-A-injected embryos (grown without 1-phenyl-2-thiourea) displayed a defective and scattered pattern of spotted pigment in the skin and eyes as compared to the Std-MO embryos (Fig. 4a and Table 1). At 2 dpf the phenotype of SMO-B morphants was remarkably similar to that of SMO-A morphants as they showed reduced body size, pericardial oedema (59%) and pigmentation defects (12%) (Fig. 4a and Table 1).

Furthermore, *in vivo* analysis at 2 dpf showed that about half of both SMO-A and SMO-B morphants had severe blood circulation defects ranging from a complete absence to reduced numbers of circulating cells (Table 1), despite a beating heart. WISH analysis of the cardiac myosin light chain 2 (*cmlc2)* expression did not show differences in the 2 dpf SMO-A and SMO-B morphants as compared to the controls, thus excluding heart morphology defects (Supplementary Fig. S1).

At 5 dpf, the majority of *usb1*-morphants never gained circulation and developed severe oedema extending to both the trunk and the yolk (Fig. 4b).

As the cardiac oedema and pigmentation defects observed in the *usb1*-morphants can be ascribed to an abnormal patterning of derivatives of the neural crest cells^19^, we explored the extent of *usb1* loss-of-function on another neural crest-specified process, chondrogenesis, by staining the cartilage of the skull and pharyngeal skeleton of 5 dpf embryos with Alcian Blue.

As shown in Fig. 4c and detailed in Table 1, 89% of Std-MO-injected larvae displayed a normal cartilages architecture. By contrast, 5 dpf embryos injected with either SMO-A or SMO-B (Fig. 4c, central and right panels) displayed either mild or severe craniofacial defects (Table 1). We consider the “mild” phenotype to be mainly characterized by abnormalities of the first two pharyngeal arches P1 (misshaped Meckel’s cartilage/lower jaw and misjoined to the palatoquadrate) and P2 (kinked and displaced ceratohyal cartilage), with grossly preserved posterior pharyngeal arches P3-P7. The “severe” phenotype was defined as reduced or absent P3-P7 bilateral branchial arches associated with the P1 and P2 defects mentioned above.

Among the SMO-A morphants, cartilage alterations were observed in 81% of the examined embryos with 31% in the mild and 50% in the severe phenotype subgroups (Table 1). Of the SMO-B morphants, 59% displayed the cartilage alterations, 27% with the mild and 32% with the severe phenotype (Table 1).

### Haematopoietic defects of *usb1*-morphants

To investigate the lineage-specific effect of *usb1*-knockdown on embryonic haematovascular morphogenesis and for further analysis of the circulation defects observed in *usb1*-morphants, we used the *tg(gata1:dsRED;flk1:GFP)* zebrafish line, which is transgenic for the red cell progenitor marker *gata1* and the vascular marker *flk1*. At 2 dpf, 62% of *tg(gata1:dsRED;flk1:GFP)* SMO-A morphants showed a reduction in *gata1*-positive cells in the context of a grossly regular vasculature pattern (Fig. 4d, Table 1).

Another transgenic line, *tg(mpx:GFP;lyzC:dsRED)*, in which the promoter of the myeloid-specific marker myeloperoxidase (*mpx*) drives the expression of GFP protein, was used to investigate neutrophils. At 2 dpf, a reduction in *mpx*-expressing cells was observed in 70% of *tg(mpx:GFP;lyzC:dsRED)* SMO-A-injected embryos (Fig. 4e, Table 1).

**Figure 5:**
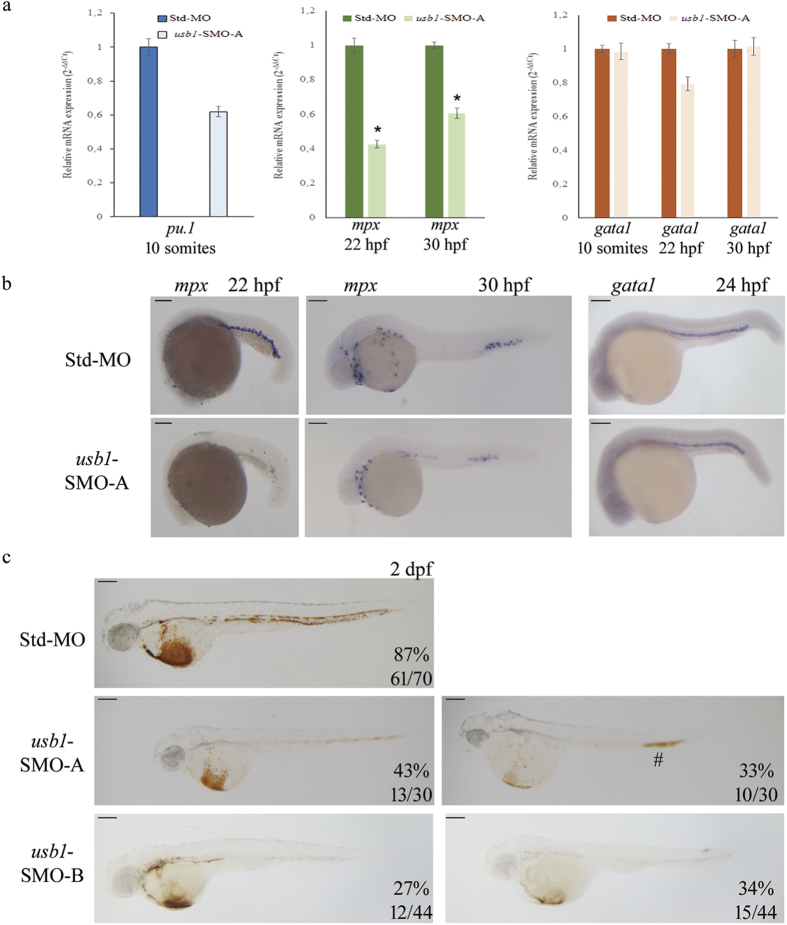
Effect of *usb1* knockdown on haematopoiesis. (**a**) Real-time PCR analysis of *pu.1*, *gata1* and *mpx* expression at the indicated developmental stages of embryos injected with SMO-A or Std-MO (each at 0.6 pmol/embryo). Samples were run in triplicate and data are expressed as the mean ± standard deviation. Asterisks (*) indicate statistically significant differences (t- test; p < 0.05). (**b**) WISH of *gata1* and *mpx* in SMO-A and control embryos at the indicated developmental stages. (**c**) O-dianisidine staining for haemoglobin in embryos injected with Std-MO (top), SMO-A (central) and SMO-B (bottom panels) (each at 0.6 pmol/embryo). The left and the right images are representative of the slight and severe erythropoiesis defects observed at the indicated percentages in SMO-A- and SMO-B-injected embryos. The # points to the caudal region of the morphant where the accumulation of blood is observed. Scale bars: (**b,c**) 200 μm.

These data prompted us to focus on the effects of *usb1*-knockdown on the primitive haematopoietic cascade in the SMO-A morphants using WISH and real-time PCR analysis. The real-time PCR analysis evidenced a substantial decrease in expression (40%) of the myeloid progenitor marker *pu.1* in 10 somites SMO-A morphants, whereas expression of the erythroid progenitor marker *gata1* was not perturbed (Fig. 5a). At 22 hpf, WISH and real-time PCR analysis showed only slightly decreased *gata1* levels (20%) (Fig. 5a,b), but at 30 hpf, *gata1*-expression levels in SMO-A and Std-MO embryos were similar (Fig. 5a).

In agreement with the data obtained with the *tg(mpx:GFP;lyzC:dsRED)* morphants, the terminally-differentiated neutrophil marker *mpx* was shown to be significantly reduced in the SMO-A-injected embryos: real-time PCR analysis showed 60% and 40% decreased expression levels of *mpx* in 22 hpf (p = 0.002) and 30 hpf morphants (p = 0.0096), respectively (Fig. 5a), fairly matching WISH results (Fig. 5b).

To determine whether red cell maturation was affected by *usb1*-knockdown, O-dianisidine staining for haemoglobin was performed. At 2 dpf, a reduction of the mature red blood cells was observed in 77% of SMO-A and 61% of SMO-B morphants, compared with a reduction in only 13% of Std-MO-injected embryos (Fig. 5c). Two subgroups could be identified: one with a slight and one with a severe reduction in haemoglobinized erythrocytes. The slightly reduced red cell phenotype was 43% for SMO-A and 27% for SMO-B morphants. A similar percentage of SMO-A (33%) and SMO-B (34%) embryos showed the severe phenotype.

### Ruling out off-target effects and validating the phenotype of *usb1* morphants

Co-injection of SMO-A and p53-MO, to check for potential off-target effects of the *usb1*-MO, resulted in a phenotype similar to that of only *usb1*-depleted embryos (Supplementary Fig. S2). *In vivo* analysis at 3 dpf showed that pericardial oedema, abnormal pigmentation and reduced circulating blood cells were all maintained (Supplementary Fig. S2).

**Figure 6:**
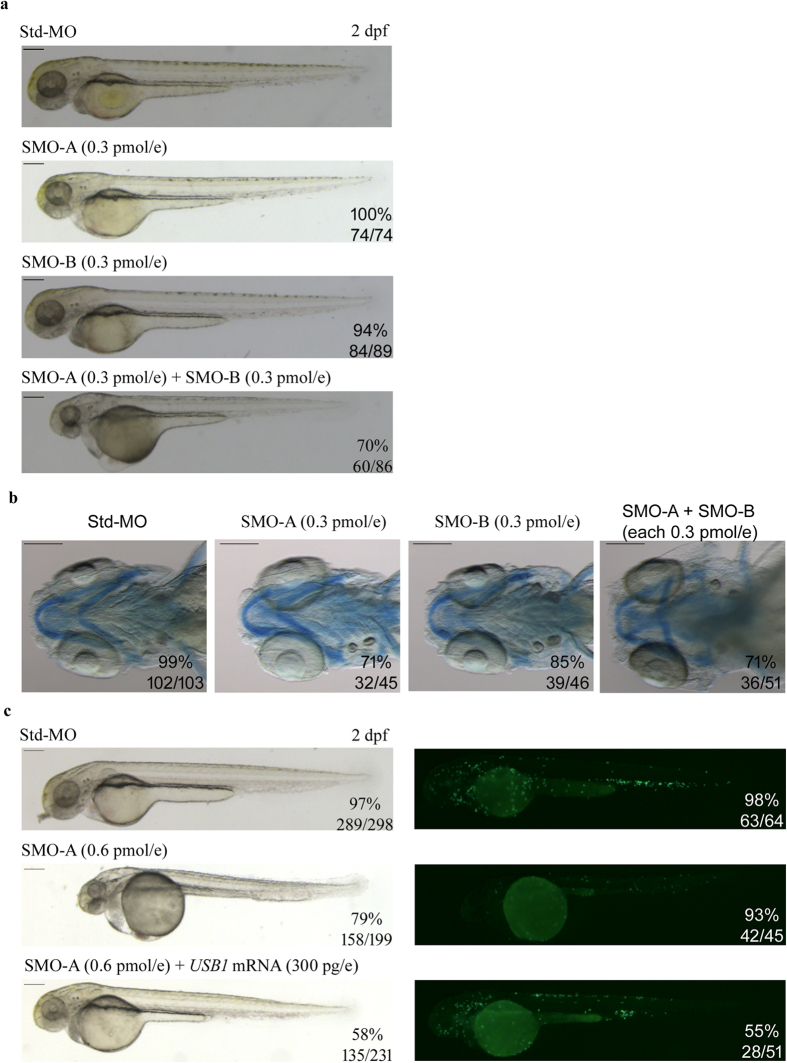
Phenotypes of morphants co-injected with SMO-A and SMO-B at subphenotypic dosage and rescue of morpholino-induced phenotypes with human *USB1* RNA. (**a**) Representative pictures of zebrafish embryos injected with Std-MO (0.6 pmol/embryo), SMO-A (0.3 pmol/embryo), SMO-B (0.3 pmol/embryo) and co-injected with SMO-A and SMO-B (each at 0.3 pmol/embryo). (**b**) Alcian blue staining at 5 dpf highlights the regular morphologic architecture of the pharyngeal arch cartilages in embryos injected with Std-MO, SMO-A and SMO-B at sub-phenotypic dosages and the aberrant cartilaginous structures in embryos co-injected with SMO-A and SMO-B (each at 0.3 pmol/embryo). (**c**) In the left panels, lateral views of 2 dpf embryos injected with Std-MO, SMO-A (0.6 pmol/embryo) and SMO-A and human *USB1* RNA (300 pg/embryo). In the right panels, fluorescent images of *tg(mpx:GFP;lyzC:dsRED)* embryos at 2 dpf. The green signals, representing *mpx*-expressing cells, are reduced in embryos injected with SMO-A (0.6 pmol/embryo) as compared to the controls, but are enhanced in embryos co-injected with human *USB1* RNA (300 pg/embryo). The fractions of embryos exhibiting the investigated phenotypes out of the total number of those examined are indicated. All images are at the same magnification. Scale bars: 200 μm.

The specificity of the *usb1* morphant phenotype was further corroborated by the co-injection of SMO-A and SMO-B at sub-phenotypic doses (each at 0.3 pmol/embryo) (Fig. 6a). When each morpholino was injected alone at this low dose, no phenotypic defects were observed. Conversely, the co-injection of both morpholinos at sub-critical doses induced an abnormal phenotype that was comparable to the one observed in the morphants injected with 0.6 pmol/embryo of SMO-A or SMO-B alone. *In vivo* analysis at 2 dpf showed that 56% of double-injected morphants displayed a paucity of circulating cells and 70% had oedema (Fig. 6a), and Alcian blue staining at 5 dpf revealed that 71% of double-injected morphants had a defective architecture of pharyngeal arches respect to controls and to embryos injected with SMO-A or SMO-B at sub-phenotypic doses (Fig. 6b).

Rescue experiments were performed on the *tg(mpx:GFP;lyzC:dsRED)* line to further confirm the specificity of the *usb1* MO-induced phenotype. Co-injection of human wild-type *USB1* mRNA (300 pg/embryo) together with SMO-A (0.6 pmol/embryo) rescued the *usb1* morphant phenotype (Fig. 6c). At 2 dpf, 58% of co-injected embryos were similar to the controls and had a normal circulation, at difference of SMO-A injected embryos, of which only a small proportion (21%) was without defects in body structure and circulation (Fig. 6c, left panels).

An increase of *mpx*-positive cells could also be appreciated in 55% of embryos co-injected with *USB1* RNA (300 pg/embryo) and SMO-A (0.6 pmol/embryo) (Fig. 6c, right panels), indicating recovery of the number of mature neutrophils.

## Discussion

In this study we showed that the developmental abnormalities observed in patients affected by Poikiloderma with Neutropenia syndrome can be effectively recapitulated in zebrafish through the knockdown of the single *usb1* gene. The two exploited splice-blocking MOs were both capable of giving rise to the signs of the human clinical spectrum, although SMO-A showed a higher penetrance of the abnormal phenotypic traits than SMO-B.

PN has two hallmarks: “poikiloderma” and mild to severe “neutropenia”. The latter predisposes to myelodysplasia and acute myeloid leukaemia^3^,^4^,^5^,^6^,^12^,^13^ thus featuring PN as a disorder of the haematopoietic cascade, mainly of the myeloid lineage. Biochemical studies on yeast and lymphoblastoid cells from PN patients have shown that *USB1* plays a crucial role in the stability and recycling of U6 snRNA, a component of the RNA splicing machinery^16^,^17^,^18^. As mutations affecting spliceosomal genes that result in defective splicing delineate a new leukaemogenic pathway^20^,^21^, the defective function of *USB1* could take part in this pathomechanism. However, splicing defects could be observed only in the yeast model, not in PN lymphoblastoid cell lines, leaving the pathogenesis of PN incompletely understood^22^.

In line with the substantial evidence that the zebrafish is an excellent system for the study of haematopoiesis during development^23^, our model, together with that recently generated by Patil *et al*.^24^, demonstrate that the *usb1* gene has a key role in normal haematopoiesis and, when dysfunctional, leads to significant reduction of mature *mpx*-neutrophils, making it a candidate for a leukaemia-causing gene.

Both our SMO-A and SMO-B morphants engendered haematological defects, as shown by the significantly reduced expression of *mpx* in the *tg(mpx:GFP;lyzC:dsRED)* morphants. Furthermore, by injecting SMO-A into the *tg(gata1:dsRED;flk1:GFP)* line, it was possible to clarify cell lineage relationships and establish that the observed reduction in blood flow was due to haematopoietic deficiency rather than a vascular defect. Parallel analysis of haematopoiesis-specific markers by WISH and real-time PCR demonstrated that the *usb1* deficit impacted on the myeloid precursors traced by the *pu.1* marker in 10 somites embryos, and on the downstream mature neutrophils, as revealed by the dramatic down-regulation of *mpx* expression in 22 hpf and 30 hpf morphants. This finding attests that the myeloid is the most severely affected lineage by *usb1* knockdown during primitive haematopoiesis. There is a parallel between the neutropenia seen in the human syndrome, which clinically is revealed by recurrent infections, and the reduction in *mpx*-positive neutrophils in zebrafish cells that have been shown to have anti-inflammatory properties^25^. Furthermore, the effect of *usb1* deficiency also affected the erythroid lineage, as mature red cells were reduced in number in 2 dpf SMO-A and SMO-B morphants. The expression levels of the erythroid progenitor marker *gata1* were variable over time: a decreased expression was observed by WISH and real-time PCR analysis in the 22 hpf morphants and was documented *in vivo* in 2 dpf *tg(gata1:dsRED;flk1:GFP)* SMO-A morphants. Conversely, real-time PCR analysis of *gata1* at 10 somites and 30 hpf showed levels of expression comparable to those in control embryos.

Taken together, our haematological results indicate the involvement of *usb1* early enough in the haematopoietic cascade to affect erythro-myeloid precursors which are present prior to the emergence of multi-lineage haematopoietic stem cells^26^, although its preeminent effect is exerted downstream of the critical bifurcation point between erythroid and myeloid lineage.

Interestingly, although almost all PN patients show involvement of the myeloid lineage, a few have been reported with multi-lineage deficiency^3^,^4^,^6^,^12^,^27^,^28^.

Other clinical signs of PN, such as poikiloderma and skeletal defects, were fully reproduced by both SMO-A and SMO-B morphants. The impact of *usb1* depletion on the zebrafish pigmentation and developing skeleton suggests that it is essential for the correct migration of ectoderm-derived neural crest-cells that differentiate in a wide spectrum of cell types, including melanocytes and craniofacial skeleton. Consistent with the physiological role of *USB1* in the morphogenesis and homeostasis of developing skin and skin annexes, our SMO-A morphants, including those generated in the two transgenic lines, displayed stripes of pigment much thinner than those in the Std-MO-injected embryos. Reduction in the clusters of spotted pigment was also observed in SMO-B morphants, in a smaller percentage and to a less degree.

Skeletal defects, including osteopenia and delayed skeletal maturation, are suggested to be more frequent in PN patients than reported, as they are mostly detectable only by X-ray^5^. The *usb1* morphants had overt and massive defects of the pharyngeal arches-derived bones, particularly those involving the Meckel’s, palatoquadrate and ceratohyal structures. The severe disruption of a few branchial arches and associated cartilages accounts for the small head of the morphants and their craniofacial defects. The leaner and smaller body of the morphants in comparison to Std-MO-injected embryos mirrors the small stature of PN patients. Interestingly, the zebrafish model of Shwachman-Bodian-Diamond syndrome (SBDS; OMIM#260400), a well characterized defect of ribosome biogenesis associated with haematopoietic dysfunction and increased cancer risk, due to mutations of the *SBDS* gene, is also characterized by skeletal defects in addition to chronic neutropenia^29^.

Moreover, the concurrence of skeletal defects, disorganized distribution of melanocytes and cardiac oedema has been observed in zebrafish with knockdown of the pinin (*pnn*) gene, which encodes a nuclear and desmosome-associated protein that modulates alternative splicing of a specific subset of target genes^30^.

The observation that SMO-A, SMO-B and co-injection of both morpholinos at sub-phenotypic doses caused a similar alteration of the overall phenotype (body size, pericardial oedema, dyspigmentation and osteocartilagineous defects) and of haematopoietic stage-specific markers (*mpx* and O-dianisidine) confirms the specificity and efficiency of the two MO-mediated *usb1*-knockdowns. Moreover, the developmental defects observed in SMO-A morphants, as the reduced body size and the paucity of neutrophils, could be almost completely rescued by co-injection with human *USB1* mRNA. All together these evidences indicate that the overall morphological and neutrophil abnormalities were caused by loss of *usb1* and support the specific effect of SMO-A and SMO-B.

The overall phenotype of the morphants co-injected with *p53*-MO and SMO-A resembled that of *usb1* morphants, suggesting that *usb1*-knockdown effects have not been caused by aspecific *p53*-activation. This result is in line with similar findings obtained with the double *sbds*- and *p53*- knockdown morphants^29^.

Finally we want to comment our data taking into account the work of Patil *et al*.^24^ who exploited as we did the morpholino knockdown technology for successful modelling Poikiloderma with Neutropenia in zebrafish. Despite differing in the main focus and the prioritised experimental tools, a few merging issues are highlighted by both studies, in particular the impact of *usb1* deficit on the neutrophil commitment and differentiation.

Indeed, while Patil and coworkers mainly explored the neutrophil specific markers, also employing the most markedly affected *ela3l* (elastase) marker for the rescue experiments, linking the significantly reduced number of neutrophils to incomplete splicing of the myeloid genes, we could monitor, by real-time PCR, the effect of defective *usb1* at the level of the myeloid progenitor through the alteration of *pu.1* expression. Our work also provides evidence on the multiple severe morphological abnormalities characteristic of the human syndrome which are associated with loss-of-function of the constitutively expressed *usb1* gene. These include pigmentation and osteochondral defects which are fairly recapitulated by SMO-A and SMO-B morphants and also by embryos co-injected with subphenotypic dosages of both morpholinos. Overall, the Japanese work and our study are well complementary and demonstrate the appropriate tool offered by the zebrafish model for further investigation of the pathogenesis of human PN syndrome.

Exploiting the zebrafish as a vertebrate model of PN, with its single highly-conserved *usb1* gene, could provide a suitable evolutionary intermediate between yeast and human to carry out further biochemichal studies aimed at clarifying the pathomechanism of PN. Targeted analysis of *usb1*-deficient zebrafish might lend insights suitable to be translated into therapies for the human disorder, particularly concerning the predisposition to myelodysplasia and acute myeloid leukaemia.

## Methods

### Zebrafish lines and maintenance

Zebrafish (*Danio rerio*) embryos were raised and maintained under standard conditions and national guidelines (Italian decree 4th March 2014, n.26). All experimental procedures were approved by IACUC (Institutional Animal Care and Use Committee).

Zebrafish AB strains obtained from the Wilson lab, University College London, London, United Kingdom and the transgenic lines *tg(gata1:dsRED;flk1:GFP)* kindly provided by the Santoro lab, Molecular biotechnology centre Università di Torino, Torino, Italia^31^, and *tg(mpx:GFP;lyzC:dsRED)* kindly provided by dr. Deflorian (IFOM Istituto FIRC di Oncologia Molecolare)^32^,^33^.

Embryos were staged according to morphological criteria^34^ and embryonic ages are expressed in somites (s), hour post fertilization (hpf) and day post fertilization (dpf). Since 24 hpf embryos were cultured in fish water containing 0,01% methylene blue to prevent fungal growth and 0,003% 1-phenyl-2-thiourea (Sigma-Aldrich, Saint Louis, Missouri, USA) to prevent pigmentation in all experiments, except those aimed at evaluating the skin phenotype.

Embryos were washed, dechorionated and anaesthetized, with 0.016% tricaine (Ethyl 3-aminobenzoate methanesulfonate salt; Sigma-Aldrich), before observations and picture acquisitions. Embryos were fixed overnight in 4% paraformaldehyde (Sigma-Aldrich) in PBS at 4 °C, then dehydrated stepwise to methanol and stored at −20 °C.

### *usb1* identification

The human USB1 amino-acid sequence was used as a query to identify in-silico the zebrafish *usb1* gene. NCBI (http://www.ncbi.nlm.nih.gov/BLAST/), ClustalW (http://www.ebi.ac.uk/Tools/clustalw/) and SMART (http://smart.embl-heidelberg.de/) tools were used for basic handling and analyses of the nucleotide and protein sequences.

Analysis of synteny was performed with Genomicus software, version 57.01 (http://www.dyogen.ens.fr/genomicus-69.01/cgi-bin/search.pl).

### Expression analysis

Total RNAs were isolated from embryos at different developmental stages and from different adult organs using the “SV Total RNA Isolation System” (Promega, Madison, Wisconsin, USA).

After treatment with DNase I RNase-free (Roche, Basel, Switzerland) to avoid possible genomic contamination, 1 μg of RNA was reverse-transcribed using the “ImProm-II™ Reverse Transcription System” (Promega) and random primers according to manufacturer’s instructions.

According to the sequence information gained from bioinformatic analysis, we amplified a fragment and the full coding sequence of *usb1* (primers F-R and F-R2, respectively; Supplementary Table S1) using GoTaq polymerase (Promega) following the manufacturer’s instructions. Specific *β-actin* primers^35^ were also used to check cDNA quality and possible genomic contamination. Reaction products were analysed by 1% agarose-gel electrophoresis. Amplicons were sequenced using Big Dye Terminator v.3.1 Cycle Sequencing Kit according to the manufacturer’s protocol on the ABI PRISM 3130 sequencer (Applied Biosystems, Foster City, California, USA). Electropherograms were analyzed with ChromasPro software 1.42 (Technelysium Pty Ltd, Tewantin QLD, Australia) using NM_001003460 as reference.

The full coding region of *usb1* was cloned into pCR™4-TOPO^®^ TA vector using “TA Topocloning for sequencing” (Invitrogen, Paisley, UK).

Sense and antisense RNA probes were respectively transcribed using T7 and T3 RNA polymerase (Roche) on templates linearized with *SpeI* or *NotI* (New England Biolabs Inc, Ipswich, Massachusetts, USA). Probes were labelled with digoxigenin using the “DIG-RNA Labelling Kit” (Roche).

WISH was performed as described^36^ with BM-Purple (Roche) as substrate. Controls with sense riboprobes were performed in parallel with antisense analyses.

Images of stained embryos were taken on a Leica MZFLIII epifluorescence stereomicroscope equipped with a DFC 480 digital camera and LAS Leica imaging software (Leica, Wetzlar, Germany).

### Morpholinos-mediated knockdown, phenotype analysis and rescue experiments

Antisense morpholinos (MOs; GeneTools, Philomath, Oregon, USA) were designed against the acceptor splice site of the *usb1* IVS2 (SMO-A, 5′–GGATCATCTGAAATTTAGGCAGGAA-3′) and IVS4 (SMO-B, 5′–CCAAGAAAAGTCCTGCGTCAACAAT-3′). A standard control oligo (Std-MO) with no target in zebrafish embryos, to check for nonspecific effects due to the injection procedure and *p53*-MO were also used according to previous reports^37^,^38^.

All morpholinos were diluted in Danieau solution^39^ and pressure-injected into 1- to 2-cell-stage embryos using Eppendorf FemtoJet Micromanipulator 5171. Rhodamine dextran (Molecular Probes, Life technology) was usually co-injected as dye tracer. The optimal dose for each MO was selected based on phenotypic effect.

RT-PCR analysis to determine efficacy of SMO splicing inhibition was carried out on RNA isolated from 2 dpf embryos using Go Taq polymerase (Promega) and F2-R3 and F-R2 *usb1* specific primers (Supplementary Table S1). Amplicons were sequenced as above mentioned.

Alcian blue and O-dianisidine staining were performed as described^40^,^41^.

Probes for *pu.1*^42^, *mpx*^25^, *gata1*^43^ and *clmc2*^44^ were synthesized and used for WISH on at least 2 batches of control and morphant embryos at each developmental stage.

Real-time PCR was performed with Fast SYBR Green Master Mix (Applied Biosystems) using the StepOne real-time PCR System (Applied Biosystems). *Actin* was chosen as endogenous normalising gene and relative expression of target genes (*mpx*, *gata1* and *pu.1*) was determined using the ΔΔCt method^45^.

Total RNA was isolated from at least three batches each of 30–40 embryos injected with *usb1*-SMO-A and Std-MO at the indicated developmental stages (10 s, 22 hpf and 30 hpf) using SV Total RNA Isolation System (Promega). All samples were retro-transcribed in two independent experiments, each RT product was run in triplicate and standard deviation (s.d.) was calculated.

Sequences of primers used for real-time PCR are listed in Supplementary Table 2.

Statistics comparisons between groups of data were evaluated using the *t* test. A p-value < of 0.05 was considered statistically significant.

Total human RNA was reverse-transcribed into cDNA using High Capacity cDNA Reverse Transcription Kit (Applied Biosystems) with random primers; then the full-length wild-type *USB1* was PCR-amplified (primer forward 5′-cggttgaggttgctggtgg-3′; primer reverse 5′-gttcctccatctcagcctg-3′). The amplicon was subcloned into the pCS2+ poly(A) vector and then used as template for generating sense RNA*, in vitro* synthesized using the “mMessage mMachine kit” (Ambion, Austin, Texas, USA) according to the manufacturer’s instructions. The synthesized mRNAs, diluted in RNase-free water, was injected into one- or two-cell-stage embryos.

## Additional Information

**How to cite this article**: Colombo, E. A. *et al*. A zebrafish model of Poikiloderma with Neutropenia recapitulates the human syndrome hallmarks and traces back neutropenia to the myeloid progenitor. *Sci. Rep*. **5**, 15814; doi: 10.1038/srep15814 (2015).

## Supplementary Material

Supplementary Information

## Figures and Tables

**Table 1:** Absolute number and percentage of each of the aberrant phenotypes observed in Std-MO, SMO-A or SMO-B injected embryos of wild-type (AB) and transgenic lines.

Frequency of morphological phenotype (%)	Std-MO	*usb1*-SMO-A	*usb1*-SMO-B
Pericardial oedema in AB line (2dpf)	34/741 (4%)	243/304 (80%)	255/433 (59%)
Abnormal pigmentation in AB line (2dpf)	0/741	71/123 (58%)	40/345 (12%)
Severely reduced circulation in AB line (2dpf)	34/741 (4%)	162/304 (53%)	205/433 (47%)
Cartilaginous defects in AB line (5dpf)	12/112 (11%)	80/99 (81%)	35/59 (59%)
Embryos *tg(gata1:dsRED;flk1:GFP)* with reduced expression of red signal	6/101 (6%)	53/85 (62%)	n.p.
Embryos *tg(mpx:GFP;lyzC:dsRED)* with reduced expression of green signal	13/93 (14%)	77/110 (70%)	n.p.

n.p. =  not performed
