# Supplementary material for: A zebrafish model of Poikiloderma with Neutropenia recapitulates the human syndrome hallmarks and traces back neutropenia to the myeloid progenitor
Source: Sci Rep. 2015 Nov 2;5:15814. doi: 10.1038/srep15814 (PMC4629135; doi:10.1038/srep15814)
Supplement: Supplementary Information [file srep15814-s1.pdf]

A zebrafish model of Poikiloderma with Neutropenia recapitulates the human syndrome hallmarks and traces back neutropenia to the myeloid progenitor

Elisa A. Colombo[1,*], Silvia Carra[2], Laura Fontana[1], Erica Bresciani[2,3], Franco Cotelli[2], Lidia Larizza[1,4]

[1] Dipartimento di Scienze della Salute, Università degli Studi di Milano, Milan, Italy.
[2] Dipartimento di Bioscienze, Università degli Studi di Milano, Milan, Italy.
[3] Oncogenesis and Development Section, National Human Genome Research Institute, National Institutes of Health, Bethesda, MD, USA.
[4] Laboratorio di Citogenetica Medica e Genetica Molecolare, IRCCS Istituto Auxologico Italiano, Milan, Italy.

*Corresponding author
Dr Elisa Adele Colombo: elisaadele.colombo@unimi.it

Key words
Poikiloderma with Neutropenia, zebrafish model, *usb1*-knockdown, defective haematopoiesis

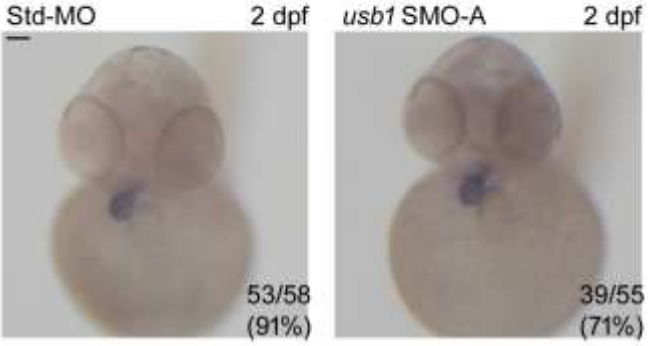

Supplementary Figure SI.
Heart morphology in usb1-depleted embryos.
WISH analysis of *cmlc2* expression showing that 2 dpf SMO-A-injected embryos display heart signals similar to those of Std-MO embryos.
Scale bar: 200 μm.

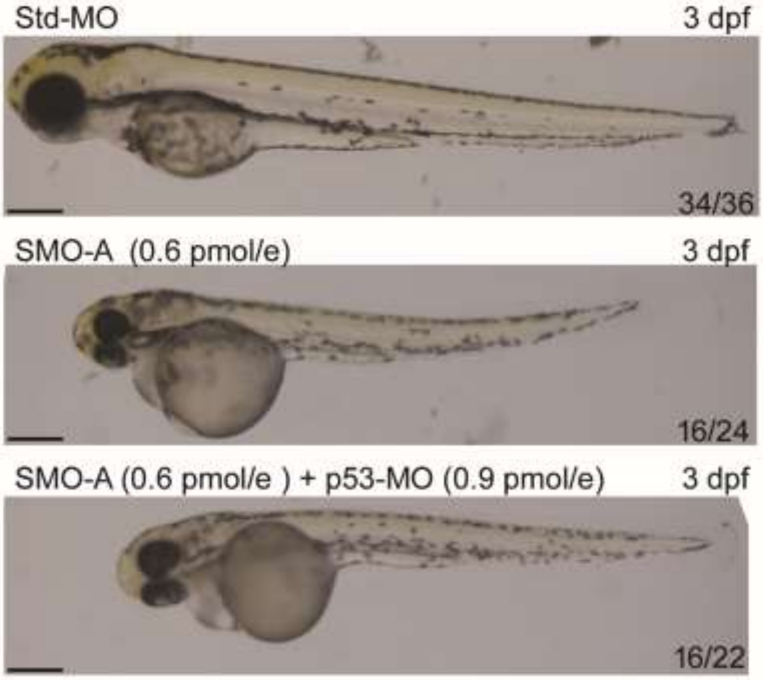

Supplementary Figure S2.
Investigation of off-target effects due to *usb1* knockdown.
The overall phenotype of co-injected p53-MO and SMO-A embryos is similar to that of SMO-A morphants. The number of embryos which phenotype is shown is indicated in each panel. Scale bar: 200 µm.

Supplementary Table 1. Specific primers used for the expression profiling of *usb1* during zebrafish development and for evaluating *usb1* knockdown efficiency.

Primer	Sequence
usb1 F	cctcgccatgattgtcaatta
usb1 F2	ccagccacttgacgcctcgtc
usb1 R	gtgtcagactggtccgaataga
usb1 R2	gtaaggcaatatgtgacctgtc
usb1 R3	tgatcccccacacaccaagcg

Supplementary Table 2. Primers for the haematopoietic markers investigated by real-time expression analysis.

Gene	Primer forward	Primer reverse	References
gata1	gagactgacctactgccatcg	tcccagaattgactgagatgag	1
mpx	tctctttttgcctgcctgat	tccattgctctcaaaccaca	This work
pu.1	gggtagccatcacatccctcta	tggacgttgtgagggtaacaca	2
actin	ccatccttcttgggtatggaat	gtcagcaatgccagggtacat	3

REFERENCES

1 Forrester, A.M. et al. NUP98-HOXA9-transgenic zebrafish develop a myeloproliferative neoplasm and provide new insight into mechanisms of myeloid leukaemogenesis. *Br. J. Haematol.* **155,** 167-181 (2011).

2 Song, H., Yan, Y.L., Titus, T., He, X. & Postlethwait, J.H. The role of stat1b in zebrafish hematopoiesis. *Mech Dev*. **128,** 442-456 (2011).

3 Gardiner, M.R., Gongora, M.M., Grimmond, S.M. & Perkins, A.C. A global role for zebrafish klf4 in embryonic erythropoiesis. *Mech Dev.* **124,** 762-774 (2007).

Supplementary Figure S1. Heart morphology in *usb1*-depleted embryos.

WISH analysis of *cmlc2* expression showing that 2 dpf SMO-A-injected embryos display heart signals similar to those of Std-MO embryos.

Scale bar: 200 μm.

Supplementary Figure S2. Investigation of off-target effects due to *usb1* knockdown.

The overall phenotype of co-injected *p53*-MO and SMO-A embryos is similar to that of SMO-A morphants. The number of embryos which phenotype is shown is indicated in each panel.

Scale bar: 200 μm.
